# The developmental regulator HAND1 inhibits gastric carcinogenesis through enhancing ER stress apoptosis *via* targeting CHOP and BAK which is augmented by cisplatin

**DOI:** 10.7150/ijbs.76345

**Published:** 2023-01-01

**Authors:** Yeye Kuang, Zhanglian He, Lili Li, Chan Wang, Xiaoqing Cheng, Qinglan Shi, Guoxiang Fu, Jianming Ying, Qian Tao, Xiaotong Hu

**Affiliations:** 1 Biomedical Research Center, Sir Run Run Shaw Hospital, Zhejiang University, Hangzhou, China.; 2 Department of Pathology, Sir Run Run Shaw Hospital, Zhejiang University, Hangzhou, China.; 3 Key Laboratory of Cancer Prevention and Intervention, Ministry of Education, Hangzhou 310016, Zhejiang, China.; 4 Cancer Epigenetics Laboratory, Department of Clinical Oncology, State Key Laboratory of Translational Oncology, Sir YK Pao Center for Cancer and Li Ka Shing Institute of Health Sciences, The Chinese University of Hong Kong, Hong Kong.

**Keywords:** HAND1, gastric cancer, tumor suppressor gene, methylation, apoptosis, CHOP, BAK.

## Abstract

Epigenetic disruption of tumor suppressor genes, particularly aberrant CpG methylation, plays a crucial role in gastric cancer (GC) pathogenesis. Through CpG methylome and expression profiling, a developmental transcription factor** -** Hand-And-Neural-crest-Derivative-expressed 1 (HAND1), was identified methylated and downregulated in GC. However, its role and underlying mechanisms in GC progression are poorly understood. Here, we show that HAND1 was frequently downregulated in GC by promoter methylation, and significantly correlated with tumor progression and poor prognosis of GC patients. High expression of HAND1 in GC patients was associated with significantly higher 5-year overall survival rates. Ectopic expression of HAND1 inhibited GC cell growth and migration* in vitro and in vivo*. HAND1 expression increased ROS levels and cytosolic Ca^2+^ concentration, enhanced cisplatin-induced apoptosis through endoplasmic reticulum (ER) stress/mitochondria-mediated apoptosis. Knockdown of CHOP and BAK attenuated HAND1-induced cell apoptosis. Overexpression of CHOP increased BAK expression. HAND1 interacts with CHOP, also directly binds to CHOP and BAK promoters and positively regulates BAK transcription. Thus, the present study demonstrates that *HAND1* is a tumor suppressor gene methylated in GC, induces ER stress and apoptosis *via* CHOP and BAK, which is augmented by cisplatin. Low HAND1 expression is an independent poor prognostic factor for GC. The tumor-specific methylation of *HAND1* promoter could be a candidate biomarker for GC.

## Introduction

Gastric cancer (GC) is the fifth most commonly diagnosed cancer and the third leading cause of cancer death worldwide. Although its overall incidence rates in northern Europe, North America, and African regions are generally low, the rates are markedly elevated in Eastern Asia, particularly in Japan, Korea and China 1, 2. In addition to microbial agents and environmental factors, the initiation and progression of GC are characterized by gradual accumulation of multiple genetic and epigenetic alterations. Epigenetic alterations are pervasive and multifaceted in GC, including DNA CpG methylation, histone modifications, RNA editing and noncoding RNAs 3, 4. Aberrant CpG methylation leads to the inactivation of tumor suppressor genes (TSGs), which then fundamentally contributes to gastric carcinogenesis and development 5, 6. Thus, identification of novel TSGs targeted by promoter methylation in GC and further exploring the related mechanisms will greatly facilitate elucidating the molecular pathogenesis of GC, as well as the development of novel effective individualized therapeutic strategies for GC patients.

We searched for novel candidate TSGs in digestive tumors through epigenomics (CpG methylome) and gene expression profiling, and discovered that a transcription factor, Hand-And-Neural-crest-Derivative-expressed 1 (HAND1), was frequently methylated and downregulated in GC. The HAND subfamily, consisting of HAND1 and HAND2, belongs to the superfamily of basic helix-loop-helix (bHLH) transcription factors. HAND factors play key roles in the regulation of cardiac, gut, and sympathetic neuronal development 7, 8. HAND factors are highly conserved across all species and activate or suppress the transcription of multiple downstream target genes 9, 10.

HAND1, located at chromosome 5q33, is essential for trophoblast giant cell differentiation and cardiac morphogenesis as a developmental regulator 11. Several previous studies have shown that HAND1 plays an important role in cell proliferation and carcinogenesis. HAND1 has been reported to be downregulated and methylated in several cancers, including colorectal, pancreatic, small cell lung, ovarian and thyroid cancers, as well as melanoma 12-19, although the underlying mechanism studies are scanty. HAND1 expression is negatively regulated by the high-mobility group A1 (HMGA1) protein, and restoration of HAND1 expression leads to reduced growth of thyroid cancer cells 18. Asuthkar *et al.* reported that nuclear translocation of HAND1 is directly regulated by uPAR protein, which controls medulloblastoma angiogenesis; HAND1 expression attenuates epithelial-mesenchymal transition (EMT) and inhibits medulloblastoma cell invasion and metastasis *via* Oct-3/4/β-catenin interaction 20-22. HAND1, which is epigenetically silenced in colon cancer, had been identified as a Polycomb target that is closely related to ES cell differentiation. Ectopic expression of HAND1 induces terminal differentiation and inhibits the growth, proliferation and xenograft tumor formation of colorectal cancer cells 16, 23. These studies indicate that HAND1 may be a tumor suppressor involved in the development of multiple cancers. However, its functions and underlying mechanisms in GC development are poorly understood.

In this study, we discovered that *HAND1* was silenced or downregulated in most GC cell lines and thus may be a TSG candidate in GC. We further investigated the inactivation of *HAND1* by promoter CpG methylation and explored its functions and potential mechanisms in the initiation and progression of GC. We found that HAND1 inhibits gastric carcinogenesis through enhancing ER stress apoptosis via targeting CHOP and BAK which is augmented by cisplatin. Moreover, HAND1 promoter methylation appears to be a good prognostic epigenetic biomarker for GC patients.

## Materials and methods

### Cell lines, tumors, and normal control tissues

A panel of GC cell lines, AGS, MKN28, MKN45, SNU1, SNU16, Kato-III, YCC1, YCC2, YCC3, YCC6, YCC7, YCC9, YCCEL1, YCC11, YCC16, and SNU719 were studied, with YCCEL1 and SNU719 as naturally EBV+ cell lines. Cell lines were purchased from ATCC or Cell Bank of Chinese Academy of Sciences and authenticated by short tandem repeat DNA profiling analysis, or from collabrators. Cell lines were cultured in RPMI-1640 or DMEM Medium (Gibco BRL, Rockville, MD) with 10% fetal bovine serum (FBS), plus 100 U/mL penicillin and 100 mg/mL streptomycin at 37 °C with 5% CO_2_.

A total of 165 GC patients who underwent surgery between May 1995 and October 2009 at the Sir Run Run Shaw Hospital (Hangzhou, Zhejiang, China) were assessed by immunohistochemistry. Patients who received preoperative radiotherapy, chemotherapy or immunotherapy before surgery were excluded from the study. Ten normal gastric mucosa biopsy samples were used as normal controls. Additionally, 35 GC cases and paired normal tissues were available for MSP. This study was approved by the ethics committee of Sir Run Run Shaw Hospital, Zhejiang University.

### Expression and methylation analyses of *HAND1* from public databases

mRNA expression analysis of HAND1 in GC specimens was retrieved from Oncomine microarray database (www.oncomine.org). Three valid mRNA expression datasets for GC in Oncomine database were analyzed. Expression data of *HAND1* were downloaded, and statistical analyses performed using GraphPad Prism version 8.0 (GraphPad Software, Inc., San Diego, CA, USA). The relevance of HAND1 methylation to mRNA expression was analyzed using online MEXPRESS (https://mexpress.be) and cBioPortal database (www.cbioportal.org).

### CpG methylome analysis

We performed CpG methylome analysis by methylated DNA Immunoprecipitation (MeDIP) using NimbleGen 385K CpG Island Plus Promoter Array, as described previously 24.

### RNA extraction, semi-quantitative RT-PCR and quantitative real-time RT-PCR

Total RNA was extracted using an RNA Kit (Omega Bio-tek, Norcross, GA). Semi-quantitative reverse-transcription PCR (RT-PCR) and quantitative real time RT-PCR (qRT-PCR) were performed with GoTaq polymerase (Promega, Madison, WI) and UltraSYBR Mixture (CWBio, Beijing, China) following the manufacturer's instructions, respectively. qRT-PCR was performed using the ABI QuantStudio 6 Flex (Applied Biosystems, Foster City, CA). GAPDH mRNA was amplified as internal control. The specific primers used in this study are listed in Table S4.

### 5-Aza-2'-deoxycytidine (5-Aza) and trichostatin A (TSA) treatment

Cells with silenced *HAND1* expression were treated with 10 μM demethylation agent, 5-Aza (5-Aza; Sigma-Aldrich, St Louis, MO) for 72 h, followed by 100 nM histone deacetylase inhibitor, TSA (TSA; Sigma-Aldrich, St Louis, MO) for 24 h^25^. After the treatment, cells were harvested for DNA and RNA extraction.

### Bisulfite treatment and promoter methylation analysis

Bisulfite modification of DNA, methylation-specific PCR (MSP) and bisulfate genome sequencing (BGS) were conducted as previously described 25, 26. MSP and BGS primers are listed in Table S4.

### Immunohistochemistry (IHC)

The ChemMate^TM^ EnVision^TM^ Detection Kit (DAKO, Carpinteria, CA) was used for IHC. Briefly, sections were incubated with HAND1 antibody (1:250 dilution; LS-B811, LSBio, Seattle, WA, USA) overnight at 4 °C.

Immunostaining results were evaluated independently by two investigators who were blinded to the clinicopathological outcomes of patients. HAND1 protein expression was scored according to the intensity of staining (0, negative; 1, weakly positive; 2, positive; and 3, strongly positive) and percentage of positive cells (0, 0-5%; 1, 5%-25%; 2, 25%-50%; 3, 50%-75%; 4, 75%-100%). The two scores were multiplied to obtain a value ranging from 0 to 12. To examine the association of HAND1 expression levels with clinicopathological features, patients were divided into two groups: low HAND1 (0-5) or high HAND1 expression (6-12).

### HAND1-expressing plasmid and cell transfection

GC cell line AGS and MKN28 were transfected with pCMV6-Entry HAND1 plasmid or empty vector (pCMV6-EntryVector) (Origene, Rockville, MD) as control, using Lipofectamine™ 3000 transfection reagent (Invitrogen, Carlsbad, CA). Stable *HAND1*-expressing and control vector clones were selected for further study.

### Cell viability assays

Stable transfected cells were seeded in 96-well plates at a density of 5 ×10^3^ cells/well and incubated for 24 h. Cell viability was assessed every day or after being treated with cisplatin (25 μM for AGS and MKN28; APExBio, Houston, TX) for 24 h according to the Cell Counting Kit-8 (CCK-8) protocol (Dojindo, Kumamoto, Japan). All experiments were performed at least in triplicate.

### Colony formation assay

For colony formation assays, 1,000 cells were seeded in a 60-mm dish and allowed to grow for 2-3 weeks. Surviving colonies (≥50 cells/colony) were counted after crystal violet staining.

### Wound healing assay

Wound healing assay was used to assess cell motility. Stably transfected cells were cultured in 6-well plates with 10 μg/mL mitomycin C (MCE, Princeton, NJ) until confluent. The cell layer was wounded using a sterile tip and washed twice with phosphate-buffered saline (PBS). Cells were incubated and photographed under a phase contrast microscope at different time points. The experiments were performed in triplicate.

### Transwell cell migration assay

For the Transwell assay, 2×10^5^ cells were resuspended in 100 μL serum-free medium and seeded in the upper chamber of a Transwell plate (Corning Inc., Corning, NY) consisting of inserts containing 8-µm pore-size PET membranes. Approximately 600 µL of medium containing 10% FBS was added to the lower chamber. After 16-24h of incubation at 37 °C, cells on the lower side of the upper chamber were fixed, stained with 0.1% crystal violet, and counted under a light microscope. The experiment was performed in triplicate.

### Cell cycle and apoptosis analysis

Cell cycle distribution and percentages of apoptosis were measured using cell cycle staining Kit (MultiSciences, Hangzhou, Zhejiang, China), Annexin V-FITC Apoptosis Detection Kit I (BD, San Jose, CA), or Annexin V-APC/7-AAD apoptosis kit (MultiSciences, Hangzhou, Zhejiang, China), with flow cytometry according to manufacturer's instructions.

### Protein extraction and Western blot

Cells were collected from cultured dishes and lysed in RIPA lysis buffer (Beyotime, Hangzhou, Zhejiang, China) supplemented with protease inhibitors or phosphatase inhibitors. Cytoplasmic, mitochondrial, and nuclear protein fractions were extracted using ProteoExtract Subcellular Proteome Extraction Kit (Millipore, Billerica, MA). Protein concentrations were quantified using a BCA Protein Assay Kit (Beyotime, Hangzhou, Zhejiang, China). Cell lysates (40 μg protein/line) were separated *via* 6%-15% SDS-PAGE and transferred to 0.45-μm thick polyvinylidene difluoride (PVDF) membranes (Millipore). The blotted membranes were blocked with 5% skim milk for 1 h at room temperature. Afterward, membranes were incubated with primary antibodies (1:1,000) overnight at 4 °C and then with HRP-labeled secondary antibody (1:2,000) for 1 h at room temperature. All the antibodies used in this study were purchased from Cell Signaling Technology (Beverly, MA). Detection was performed on a Fujifilm Las-4000 Luminescent Imaging System using ECL Kit (Pierce, Rockford, IL).

### Measurement of MMP

MMP assay kit (Beyotime) with JC-1 was used to detect MMP as described by the manufacturer. Briefly, the cells were collected and stained with 0.5 mL JC-1 working solution for 20 min at 37 °C. Then, the percentage of red and green fluorescence was estimated by flowcytometry. All experiments were replicated in triplicate.

### Measurement of intracellular Ca^2+^ levels

Intracellular Ca^2+^ levels were measured using Fluo-3AM (Beyotime) as previously described 27, 28. [Ca^2+^]i was derived after calibration according to the following equation: [Ca^2+^]i (nM)=K_d_ (F-F_min_)/(F_max_-F). F is the base line fluorescence. F_min_ is the fluorescence in the presence of EGTA. Fmax is the fluorescence detected with saturating Ca^2+^. K_d_ (400 nM) is the dissociation constant of Fluo-3AM for Ca^2+^ at room temperature. All experiments were replicated in triplicate.

### Measurement of ROS

ROS Assay Kit (Beyotime) was used to measure ROS production levels as described by the manufacturer. Briefly, 1×10^6^ cells were stained with 10 μM DCFH-DA for 20 min at 37 °C and analyzed by flowcytometry. All experiments were replicated in triplicate.

### RNA sequencing (RNA-Seq)

Total RNA was extracted from stable *HAND1*-expressing and control vector cells, replicated thrice for each sample. RNA amount and purity of each sample were quantified using NanoDrop ND-1000 (NanoDrop, Wilmington, DE). Poly (A) RNA is purified from 1 μg total RNA using Dynabeads Oligo (dT)25-61005 (Thermo Fisher, Carlsbad, CA), and fragmented into small pieces using Magnesium RNA Fragmentation Module (NEB, Ipswich, MA) under elevated temperature. Cleaved RNA fragments were reverse-transcribed and the final cDNA library was constructed according to the protocol for mRNA-Seq sample preparation kit (Illumina, San Diego, CA). Subsequently, paired-end sequencing was performed on an Illumina Novaseq™ 6000 (LC-Bio, Hangzhou, Zhejiang, China) following the vendor's recommended protocol.

Sequencing reads were aligned to human reference genome (GRCh38) using HISAT2 software (Version 2.0.4) after trimming adapter sequences and removing low-quality reads using Cutadapt software (Version 1.9). FPKM were used to estimate the relative abundance of all transcripts and expression level for mRNAs. Differentially expressed mRNAs were selected with *p* value < 0.05 and fold change > 2, or fold change < 0.5 by R package edgeR and DESeq2, and then GO enrichment and KEGG enrichment analysis were performed.

### *In vivo* subcutaneous tumor model

All *in vivo* animal experiments were approved by the animal care committee of Sir Run Run Shaw Hospital. Viable MKN28 and control cells (5 × 10^6^ cells in 100 μL PBS) were injected subcutaneously into the right dorsal flank of 6-week-old female BALB/c nude mice (five mice per group). Tumor volume was measured every two days, and tumor weight was measured at the end of the fourth week. Tumor volume was calculated using the following formula: (Short diameter)^2^ × (Long diameter)/2.

### RNA interference

Stable transfected cells were transfected with BAK siRNA (Ambion, Austin, TX), CHOP siRNA (Genechem, Shanghai, China) or negative control siRNA using Lipofectamine RNAiMax Transfection Reagent (Invitrogen) according to manufacturer's instructions. Approximately 72 h later, total proteins were extracted, and the efficiency of siRNA was confirmed by Western blotting.

### Chromatin immunoprecipitation (ChIP)

ChIP assay was performed using Simple ChIP^®^ Plus Enzymatic Chromatin IP Kit (Cell Signaling Technology, Danvers, MA). Sonicated nuclear fractions were incubated with anti-FLAG (Cell Signaling Technology), positive control histone H3 (Cell Signaling Technology), and negative control normal rabbit IgG (Cell Signaling Technology). Immunoprecipitated DNA was identified by PCR using specific primers for BAK or CHOP promoter. The sequences of the primers are shown in Table S4.

### Luciferase reporter assay

Stable transfected cells were co-transfected with CHOP promotor-firefly luciferase reporter plasmid and pRL-TK Renilla plasmid (Genechem, Shanghai, China), or negative control and BAK-GLuc (GeneCopoeia, Rockville, MD). Luciferase activity was normalized by that of cells co-transfected with pRL-TK Renilla vector or SEAP expression vector (GeneCopoeia). Supernatants of GLuc-transfected cells or cell lysates were analyzed with a Dual-Lumi™ Luciferase Reporter Gene Assay Kit (Beyotime) or Secrete-Pair™ Dual Luminescence Assay Kit (GeneCopoeia) according to manufacturer's protocols.

### Immunofluorescence

Cells grown on coverslips were fixed with 4% paraformaldehyde (Beyotime) for 10 min, permeabilized in 0.1% Triton X-100 for 4 min and blocked with 3% bovine serum albumin (Beyotime) for 20 min. Cells were subsequently incubated at 4°C overnight with HAND1 monoclonal antibody (Origene), or FLAG Tag antibody (Cell Signaling Technology) and CHOP polyclonal antibody (Proteintech, Chicago, IL), and incubated with both anti-mouse Alexa-Fluor 488 and Rhodamine phalloidin (Invitrogen) or anti-rabbit Alexa-Fluor 568 (Invitrogen) for 1 h at room temperature. Rhodamine phalloidin staining was used to visualize F-actin. Nuclei were counterstained with 4,6-diamidino-2-phenylindole (DAPI, Invitrogen), and fluorescence examined by Olympus BX51 microscope (Olympus, Tokyo, Japan).

### Co-immunoprecipitation (Co-IP) assay

Total protein lysates were extracted from cells using RIPA lysis buffer (Beyotime) supplemented with protease inhibitors cocktail. 500 μg protein lysates, combined with 10 µg of CHOP polyclonal antibody (Proteintech), were incubated for 1-2 h at room temperature with mixing. Then, 25 µL Protein A/G magnetic beads (Thermo Fisher) were added and the mixture was incubated at 4 °C overnight with mixing. The obtained immune complexes were washed four times, boiled with 2× SDS-PAGE Sample Loading Buffer (Beyotime), and subsequently detected by Western blot.

### Statistical analysis

Results were all presented as mean values ± SD. Statistical analyses were performed in SPSS version 19.0 (SPSS Inc., Chicago, IL) and GraphPad Prism software version 8.0 (GraphPad Software Inc., San Diego, CA). One-way ANOVA or two-tailed Student's t-test were used to analyze differences between groups. Chi-square tests were used to analyze the relationship between *HAND1* expression and clinicopathological parameters. OS was calculated using Kaplan-Meier method with log-rank test. Univariate and multivariate Cox regression analyses were performed to evaluate prognostic factors. For all tests, *p*<0.05 was considered statistically significant.

## Results

### Expression and CpG methylome study identifies *HAND1* as a methylation-silenced target in gastric cancer

**Figure 1:**
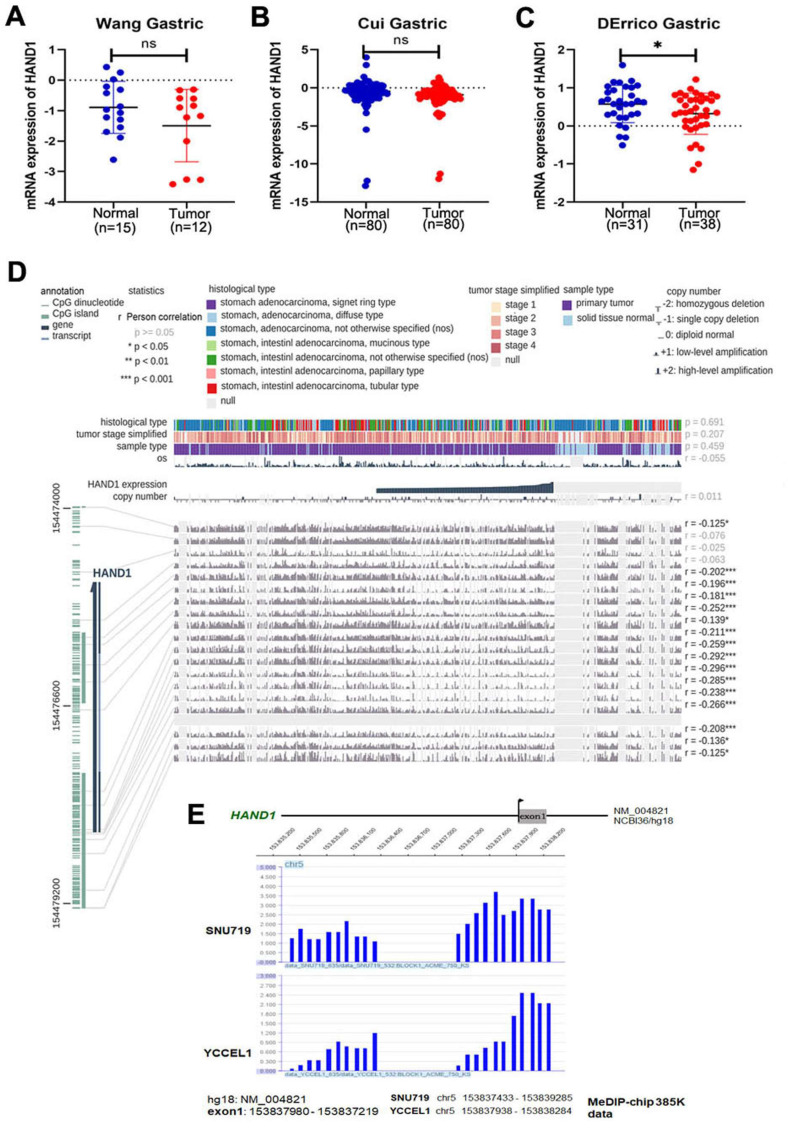
** GC methylome study identifies *HAND1* promoter methylation in gastric cancer and associated with its downregulation. (A-C)** *HAND1* mRNA expression is frequently downregulated in gastric tumor cases (Tumor) compared with normal gastric samples (Normal) in Oncomine database. **(D)** *HAND1* expression is negatively correlated with promoter CpG methylation, by Pearson correlation coefficients in MEXPRESS database. **(E)** CpG methylome analysis by MeDIP-Chip demonstrate signal enrichment in *HAND1* promoter CGI in gastric cancer. Positive signal peaks (blue) are marked. Data is presented as mean ± SD. ^*^p<0.05; ^**^p<0.01; ^***^p<0.001.

Through analyzing *HAND1* mRNA expression data of GC patients in the Oncomine database, we found that *HAND1* was downregulated in GC cases compared with normal gastric samples (Figure 1A-C). To further determine the mechanism of *HAND1* downregulation in GC, we analyzed the correlation between *HAND1* expression and CpG methylation in TCGA stomach adenocarcinoma dataset using MEXPRESS. Assessment using Pearson's correlation coefficient indicated that *HAND1* expression in GC is significantly negatively correlated with its methylation levels (r up to -0.296, *p*<0.001, Figure 1D), indicating that promoter methylation might lead to *HAND1* mRNA downregulation. Meanwhile, we performed CpG methylome analysis to identify cancer genes in GC. Methylome data showed strong signal enrichment in CpG island (CGI) of *HAND1* promoter in SNU719 and YCCEL1 gastric cell lines, and defined *HAND1* as a methylated target in GC (Figure 1E).

### *HAND1* silencing/downregulation by promoter methylation in GC cell lines and tumors

**Figure 2:**
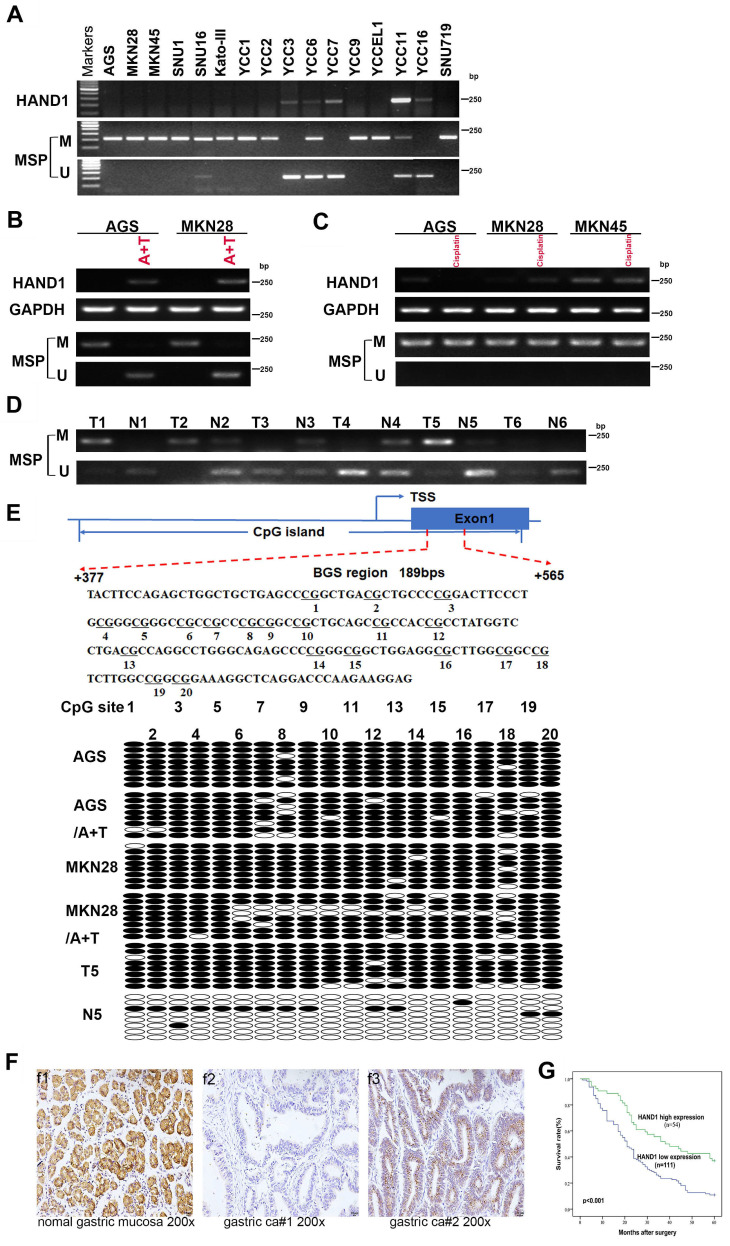
***HAND1* expression and methylation status in GC cell lines and primary tumors, and its prognosis value. (A)** Silencing or downregulation of *HAND1* in GC cell lines due to its promoter methylation. **(B)** RTPCR and MSP show that *HAND1* in silenced and methylated GC cell lines, but restored after treatment with demethylation agent 5-Aza and TSA (A+T). Representative results are shown. **(C)** MSP analysis shows that HAND1 promoter methylation or mRNA expression in silenced and methylated GC cell lines was not affected by treatment with Cisplatin for 24 h (25 μM). **(D)** Representative results of *HAND1* promoter methylation by MSP in primary gastric tumor tissues (T) and paired adjacent normal tissues (N). **(E)** Representative BGS results on the methylation status of *HAND1* CGI. Cloned BGS-PCR products are sequenced, and each colony shown as an individual row, representing a single allele of the CGI. Open circles represent unmethylated, and filled circles represent methylated CpG sites. **(F)** Representative immunohistochemical staining of HAND1 in normal gastric mucosa and GC tissues. Original magnification: 200×. **(G)** Kaplan-Meier survival analysis about the relationship of *HAND1* expression and five-year overall survival rates in GC patients. GC patients with low *HAND1* expression had poor prognosis. M: methylated; U: unmethylated. TSS: transcriptional start site.

To further validate *HAND1* expression and methylation status in GC tumors, we assessed *HAND1* mRNA expression in GC cell lines. Results showed that *HAND1* expression was silenced or downregulated in most cell lines (Figure 2A). Then, methylation-specific PCR (MSP) was conducted to analyze *HAND1* promoter methylation status. *HAND1* methylation was observed in 13/16 (81%) of cell lines (Figure 2A).

Meanwhile, *HAND1* mRNA expression was restored after demethylation treatment with 5-Aza and TSA, and representative results are shown in Figure 2B. Promoter methylation and mRNA expression of *HAND1* were not affected by treatment with Cisplatin (Figure 2C). Detailed methylation profiling of *HAND1* CGI was further performed by bisulfite genomic sequencing (BGS) analysis of 20 CpG sites in the CGI (Figure 2E). BGS showed that *HAND1* CGI was heavily methylated in GC cell lines, whereas only partial demethylation detected after 5-Aza and TSA treatment, in agreement with the MSP results (Figure 2B, 2E).

We further investigated *HAND1* promoter methylation in 35 pairs of GC tissue samples by MSP. In 60% (21/35) of cases, *HAND1* methylation levels were higher than paired adjacent normal controls. Similar results were obtained by BGS analysis of paired GC and adjacent normal tissues. Representative results are shown in Figure 2D and 2E. These results indicate that *HAND1* expression is regulated through promoter CGI methylation.

### Relationship of HAND1 expression and clinicopathological features of GC patients

HAND1 protein expression was evaluated by immunohistochemistry in 10 normal gastric mucosa biopsy specimens and 165 gastric cancer cases. HAND1 protein was mainly expressed in the cytoplasm and nuclei of tissues (Figure 2F). HAND1 protein was highly expressed in all normal gastric mucosa, but absent or downregulated in 67 % (111/165) of GC samples.

Correlation between clinicopathological parameters and HAND1 protein expression of GC patients was further analyzed (Table S1). HAND1 expression was significantly correlated with gender (*p*=0.019), histopathological grading (*p*=0.050), depth of invasion (*p*=0.018), lymph nodal status (*p*=0.017), and TNM stage (*p*=0.005). To ascertain the effect of HAND1 expression on the prognosis of GC patients, all GC patients were followed up for five-year overall survival (OS) after surgery. Kaplan-Meier survival analysis showed that GC patients of high HAND1 expression had higher five-year overall survival rates, compared with those with low HAND1 expression (*p*<0.001, Figure 2G).

Univariate and multivariate analyses with Cox regression were conducted to determine key prognostic factors of OS (Table S2 and S3). In univariate analysis, HAND1 expression (*p*<0.001), age (*p*=0.012), histopathological grading (*p*=0.047), depth of invasion (*p*<0.001), lymph node metastasis (*p*<0.001), distant metastasis (*p*<0.001), and TNM stage (*p*<0.001) were significantly associated with OS in GC patients. These statistically significant factors were introduced to the Cox regression model, and multivariate analyses were conducted. The results showed that HAND1 expression (*p*<0.001), distant metastasis (*p*= 0.0046), and depth of invasion (*p*<0.001) were independent prognostic factors for OS in GC. These results suggested that low expression of HAND1 predicted poor prognosis and was correlated with tumor progression in GC patients.

### Ectopic HAND1 expression inhibits GC cell growth and migration

**Figure 3:**
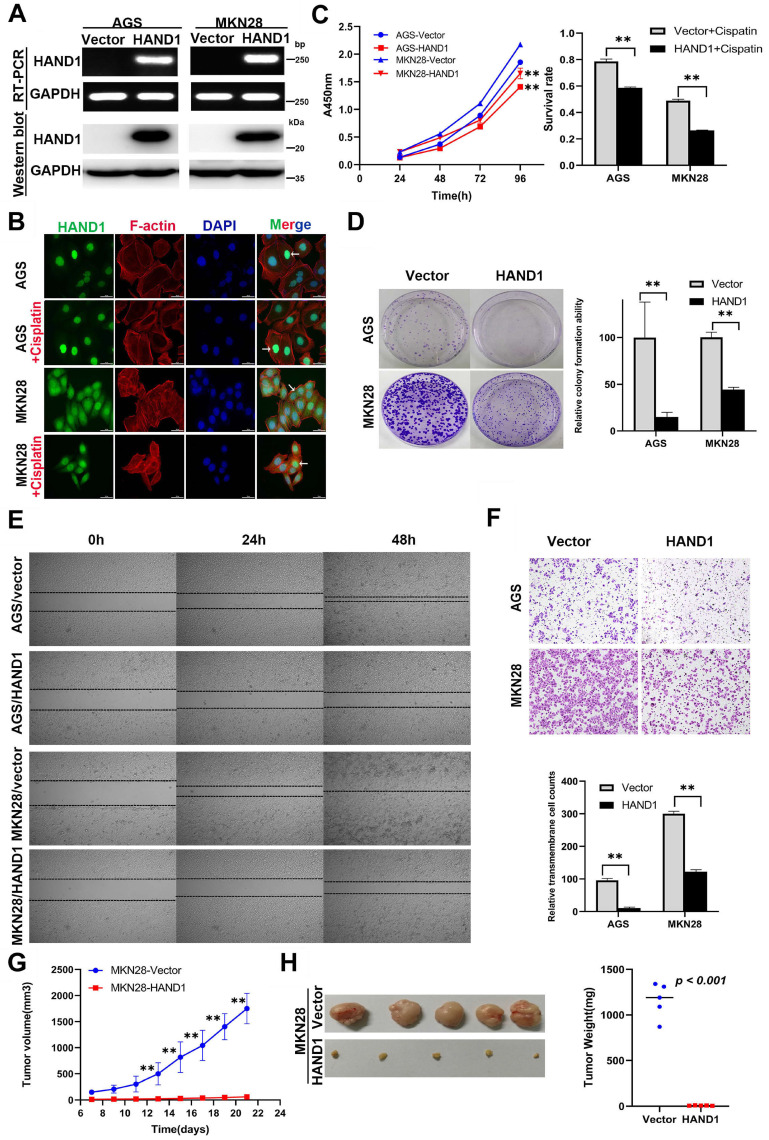
** Ectopic *HAND1* expression inhibits GC cell growth and migration. (A)** HAND1 mRNA and protein expression in stably transfected cells as confirmed by RT-PCR and Western blot. **(B)** Immunofluorescence staining is used to identify the subcellular location of HAND1 in HAND1-transfected cells before and after cisplatin treatment for 24 h (25 μM). Scale bar, 10 µm. **(C)** HAND1 significantly inhibits cell viability, without or with cisplatin treatment. **(D)** HAND1 significantly inhibits cell colony formation. **(E)** Representative photos of wound healing assay (Original magnification:100×). **(F)** Representative images of Transwell assays (Original magnification:100×). The number of migrating cells in five random fields per Transwell was counted for quantitative analysis. **(G-H)** HAND1 suppresses subcutaneous tumor growth in nude mice. Quantitative analyses of tumor volume and tumor weight are shown. All values are expressed as mean ± SD of three independent experiments. ^*^p<0.05; ^**^p<0.01.

*HAND1* downregulation by promoter methylation was significantly associated with malignant progression of GC, indicating its important role in GC tumorigenesis, we thus further investigated its potential biological functions in GC cells. We established two cell lines (AGS and MKN28) stably-expressing *HAND1*, with empty vector transfection as control. Expression levels of *HAND1* mRNA and protein in these cell lines were confirmed by RT-PCR and Western blot (Figure 3A). Immunofluorescence assays showed that HAND1 protein was predominantly localized in the nucleus (Figure 3B). Cell viability assays showed that the cell survival rate of *HAND1*-transfected cells was significantly lower than that of control cells, especially after cisplatin treatment (*p*<0.05, Figure 3C). Moreover, ectopic *HAND1* expression significantly inhibited the colony formation ability of transfected cells, compared with control cells (*p*<0.05, Figure 3D).

To assess the effects of *HAND1* expression on the migration and invasion of GC cells, wound healing and transwell assays were conducted. Wound-healing assays showed that *HAND1* stably-expressing cells took longer to heal the wound than that of control cells (Figure 3E). Transwell assays also showed that HAND1 inhibits GC cell migration (*p*<0.05, Figure 3F).

We also evaluated the effects of HAND1 on GC cell growth *in vivo* by injecting MKN28-HAND1 cells into nude mice. The tumor growth rate of MKN28-HAND1 cells in nude mice was significantly lower than that of control cells (*p*<0.05, Figure 3G). Tumor weight was significantly lower in *HAND1*-expressing nude mouse group, compared with control group (*p*<0.05, Figure 3H). These results suggested that HAND1 inhibits GC cell growth and migration *in vitro* and *in vivo,* and functions as a tumor suppressor in GC carcinogenesis.

### Ectopic HAND1 expression upregulates apoptosis and intracellular reactive oxygen species levels

**Figure 4:**
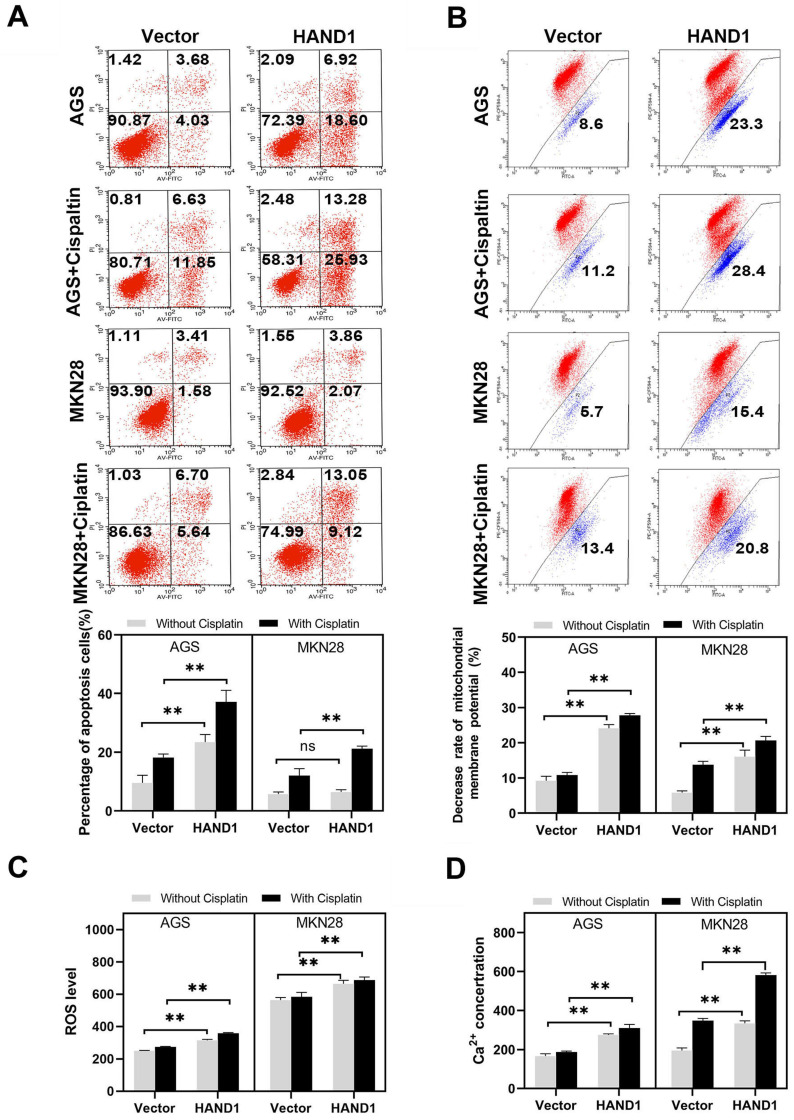
** Effect of HAND1 on apoptosis, mitochondrial membrane potential, ROS and cytosolic Ca^2+^ concentration in GC cells. (A)** Representative result and quantitative analysis of Annexin V-FITC/PI staining in stably transfected GC cells, before and after cisplatin treatment. **(B)** HAND1 significantly decreases mitochondrial membrane potential. Representative results and quantitative analysis are shown. **(C)** Analysis of ROS generation in stably transfected GC cells. **(D)** Effect of HAND1 on the variation of cytosolic Ca^2+^ concentration in GC cells. All values are presented as mean ± SD of three independent experiments. ^*^p<0.05; ^**^p<0.01.

To investigate whether inhibition of GC cell proliferation and growth by HAND1 is related to cell apoptosis and cell cycle arrest, apoptosis and cell cycle analyses were conducted. Cell cycle analysis showed that the cell cycle distribution of *HAND1*-transfected cells was not significantly different from that of control cells (data not shown). We then examined spontaneous and cisplatin-induced apoptosis of *HAND1*-transfected cells and control cells. *HAND1* expression significantly increased the spontaneous apoptosis in AGS, but not in MKN28. After cisplatin treatment, the percentage of apoptosis cells in *HAND1*-transfected AGS and MKN28 cells was significantly higher, compared with control cells (*p*<0.05, Figure 4A). To further determine the mechanism of apoptosis promotion by HAND1, we assessed the mitochondrial membrane potential (MMP), intracellular reactive oxygen species (ROS) levels, and cytosolic Ca^2+^ concentrations. A decrease in MMP is a near sign of cell apoptosis. Our results showed that MMP in *HAND1*-transfected cells with or without cisplatin treatment significantly decreased compared to control cells (*p*<0.05, Figure 4B). Moreover, *HAND1*-transfected cells had significantly higher intracellular ROS levels than control cells, regardless of cisplatin treatment (*p*<0.05, Figure 4C). Similarly, *HAND1*-transfected cells possessed higher cytosolic Ca^2+^ concentrations compared with control cells, especially when treated with cisplatin (*p*<0.05, Figure 4D).

### HAND1 induces ER stress-mediated apoptosis and enhances mitochondria-mediated apoptosis

**Figure 5:**
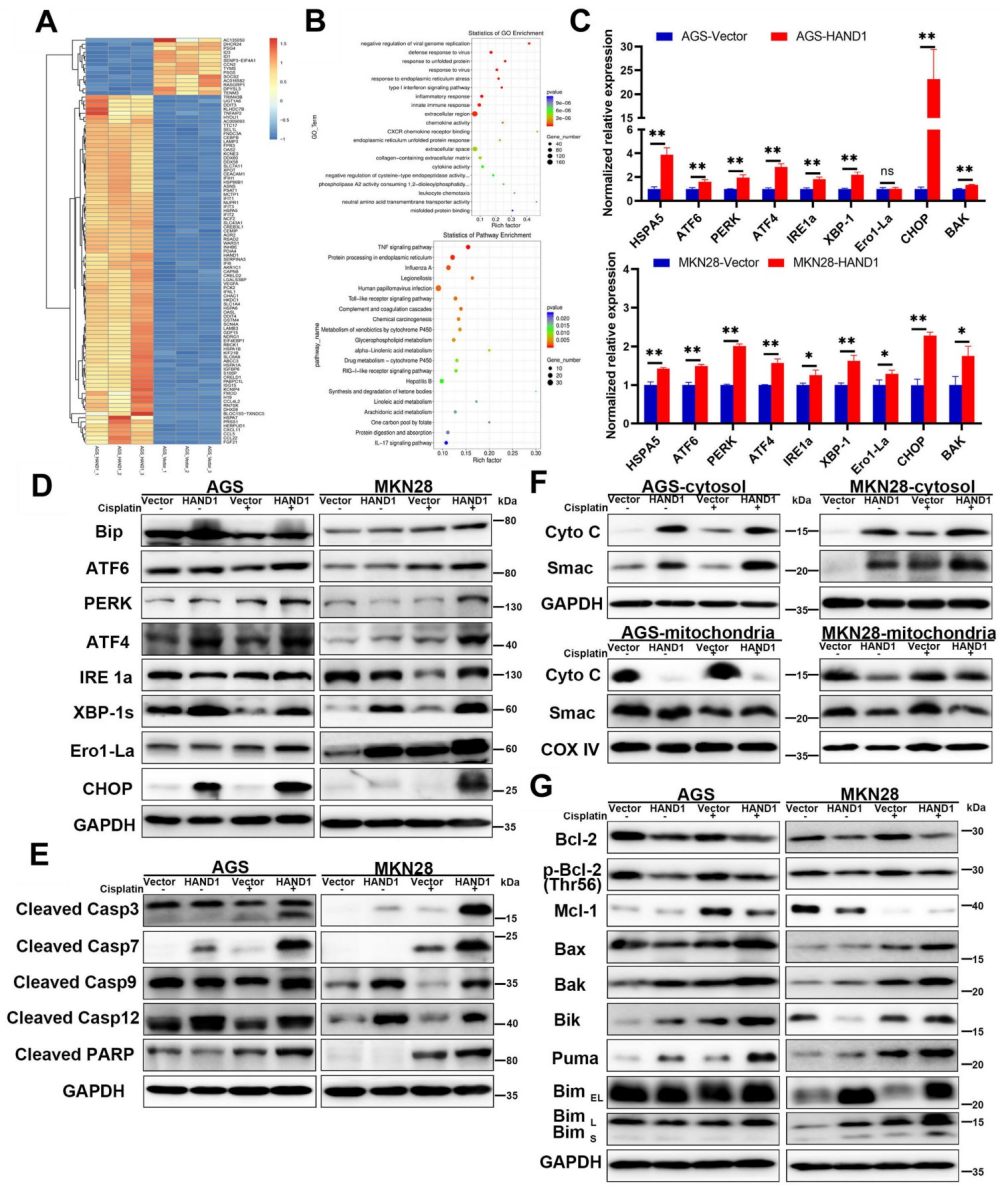
** HAND1 induces ER stress-mediated apoptosis *via* the mitochondria apoptosis pathway. (A)** Clustered heatmap view using the top 100 differentially expressed genes (DEGs) with the lowest P values from triplicate samples of HAND1-transfected AGS cells and control cells. The color ranged from blue to white to red indicates the level of gene expression ranged from low to high. **(B)** Bulb map of GO analysis and KEGG analysis of the DEGs.** (C)** Quantitative real-time RT-PCR (qRT-PCR) to confirm RNA-Seq results. Two up-regulated genes (HSPA5 and CHOP) from the clustered heatmap, and seven genes related to ER stress-mediated apoptosis were examined.** (D)** Several key UPR-related proteins were checked by Western blotting of stably transfected GC cells.** (E)** Cleaved caspase-3, caspase-7, caspase-9, caspase-12 and PARP are upregulated by HAND1 expression. **(F)** HAND1 increases the release of Cyto C and Smac from mitochondria into cytosol. **(G)** Several key BCL-2 family proteins checked by Western blotting in stably transfected GC cells.

The above results predicted that HAND1 triggers ER stress-mediated apoptosis and subsequently mitochondria-mediated apoptosis. To verify that further, we carried out RNA-seq, focusing on the top 100 differentially expressed genes (DEGs) with the lowest *P* values for *HAND1*-transfected AGS cells and control cells (Figure 5A). Gene ontology (GO) and KEGG pathway enrichment analyses showed that several enriched terms were correlated with ER functions, such as “response to unfolded protein”, “response to ER stress”, “ER unfolded protein response”, and “Protein processing in ER” (Figure 5B). Consistent with this result, qRT-PCR and Western blotting analyses showed that *HAND1* expression upregulated a series of ER-stress- and unfolded protein response (UPR)- related genes, including HSPA5 (also known as Bip), CHOP (also known as DDIT3), ATF6, PERK, ATF4, IRE1a, XBP-1s, and Ero1-La, especially after cisplatin treatment (Figure 5C-D). We also detected the expression of some apoptosis-related proteins and found that *HAND1* expression increased the cleavage of caspase-3, caspase-7, caspase-9, caspase-12 and PARP, especially after cisplatin treatment (Figure 5E). These results suggest that HAND1 promotes ER stress-induced apoptosis and activates signaling pathways, including UPR in GC cells.

We further investigated whether HAND1 affected mitochondria-mediated apoptosis. We isolated the mitochondria and cytosol fractionation in *HAND1*-expressing cells, and found that the release of cytochrome c (Cyto C) and Smac, two indicators of mitochondria-mediated apoptosis, from mitochondria into cytosol, was increased in *HAND1*-transfected cells compared to control cells (Figure 5F). Proteins of the BCL-2 family are central regulators of mitochondria-mediated apoptosis, so some anti-apoptotic proteins and pro-apoptotic proteins were assessed. Our results showed that *HAND1* expression decreased anti-apoptotic protein Bcl-2, p-Bcl-2 (Thr56), and Mcl-1 expression, and increased pro-apoptotic protein Bax, Bak, Bik, Bim, and Puma expression (Figure 5G). In addition, HAND1 expression upregulated *BAK* mRNA expression (Figure 5C). These findings suggested that HAND1 also promotes mitochondria-mediated apoptosis by regulating BCL-2 family proteins in GC cells.

### HAND1 interacts with CHOP, targets CHOP and BAK promoter, and promotes GC cell apoptosis

**Figure 6:**
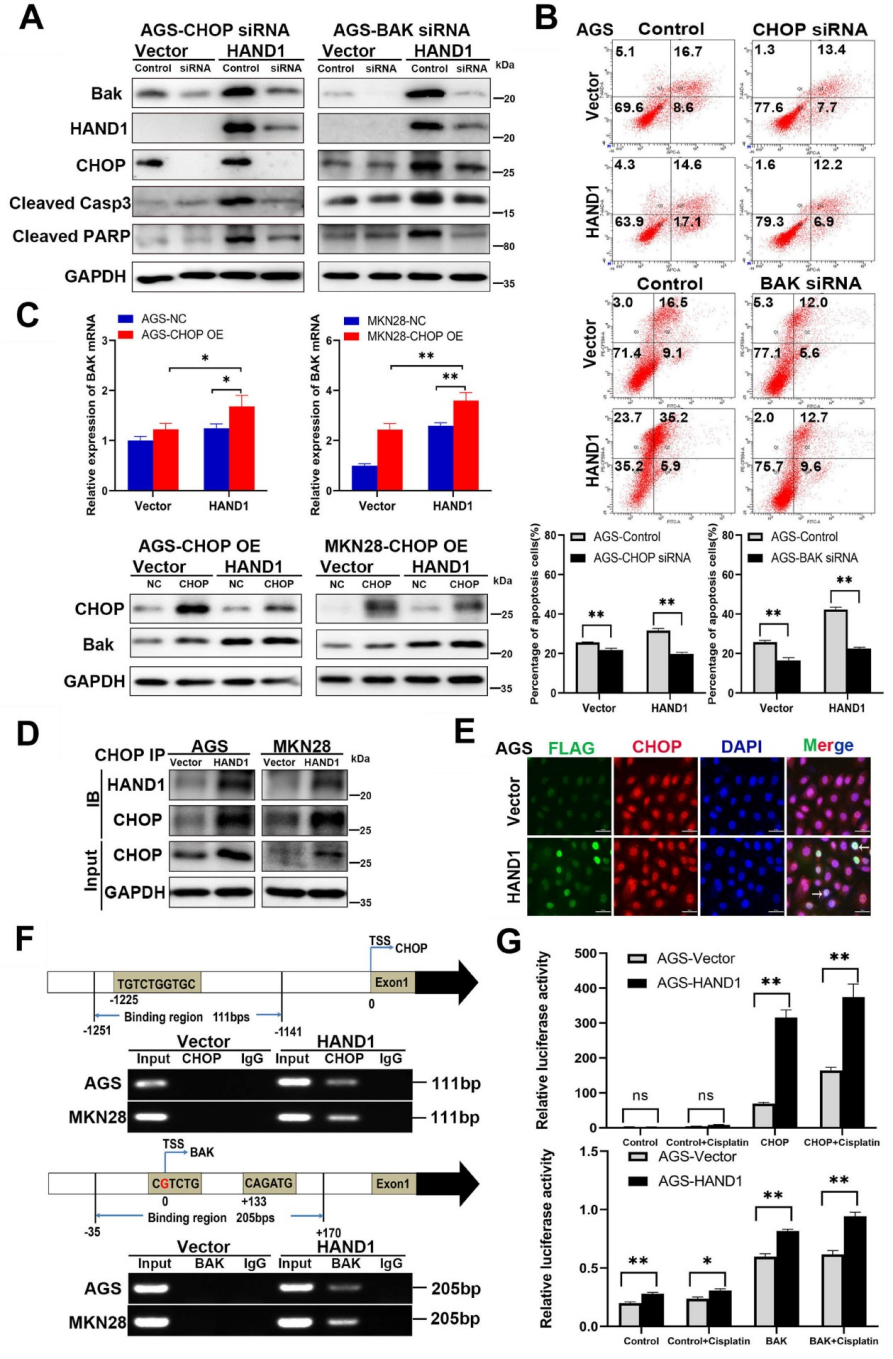
** HAND1 interacts with CHOP, targets CHOP and BAK promoters and upregulates their expression and further resulting in GC cell apoptosis. (A)** Knockdown of CHOP or BAK decreases Bak, HAND1, CHOP, cleaved caspase-3, and cleaved PARP in HAND1-transfected AGS cells compared with controls. **(B)** Representative results and quantitative analysis of apoptosis in stably transfected AGS cells with CHOP or BAK knockdown after cisplatin treatment. **(C)** Ectopic expression of CHOP increases the expression of BAK mRNA and protein in HAND1-transfected cells. **(D)** Co-IP assay examines the interaction between HAND1 and CHOP in stably transfected GC cells. **(E)** Immunofluorescence detects the interaction between HAND1 and CHOP in stably transfected AGS cells. Scale bar, 10 µm.** (F)** Binding of HAND1 to CHOP and BAK promoters as assessed by ChIP. IgG is used as a negative control, and Input as a positive control. **(G)** HAND1 binds to CHOP and BAK promoters and regulates their transcription in *HAND1*-transfected AGS cells. Data are presented as mean ± SD of three independent experiments. ^*^p<0.05; ^**^p<0.01.

The above results showed that HAND1 promotes ER stress-mediated and mitochondria-mediated apoptosis by activating UPR and regulating BCL-2 family proteins, especially upregulation of the hallmark genes CHOP and BAK. To further determine its relationship with CHOP and BAK, we silenced *CHOP* or *BAK* with specific siRNA or overexpressed CHOP, and further investigated the effects of HAND1 on GC cell apoptosis. The efficiency of *CHOP* and *BAK* knockdown was confirmed by Western blotting. HAND1 induced upregulation of CHOP and BAK protein levels. However, after knockdown of *CHOP* or *BAK*, apoptosis markers - cleaved caspase-3 and cleaved PARP markedly decreased in *HAND1*-transfected cells, indicating decrease of cell apoptosis (Figure 6A and S1A). Knockdown of *CHOP* or *BAK* also resulted in decreased HAND1 levels, indicating a possible feedback loop of HAND1 regulation.

After cisplatin treatment for 24h, the percentage of apoptotic cells significantly decreased in *HAND1*-transfected cells with *CHOP* or *BAK* knockdown, compared to controls (*p*<0.05, Figure 6B and S1B). Overexpression of *CHOP* caused significantly upregulation of *BAK* mRNA and protein expression in *HAND1*-transfected cells (*p*<0.05, Figure 6C). In addition, co-IP and immunofluorescence assays revealed an interaction of HAND1 and CHOP proteins (Figure 6D-E and S1C), suggesting that HAND1 could interact with CHOP to regulate its target genes and promotes cell apoptosis *via* CHOP and BAK.

Previous studies showed that HAND1 binds to DNA with a consensus sequence “NNTCTG” 29, 30. We hypothesize that HAND1 binds to CHOP or BAK promoters and regulates their transcription. We indeed found binding sites containing “NNTCTG” in CHOP or BAK promoters through analyzing JASPARCORE and TRANSFAC databases. Our further ChIP assays showed that HAND1 binds directly to CHOP promoter (nt -1251 to -1141 related to the transcription start site (TSS)) and BAK promoter (nt -35 to +170 related to TSS) (Figure 6F). To further investigate the effects of HAND1 binding to CHOP or BAK promoter, luciferase reporter assays were conducted. The result showed that their promoter luciferase activity was both markedly increased in HAND1-transfected cells, with or without cisplatin treatment, compared with control cells (*p*<0.05, Figure 6G and S1D), consistent with our previous finding of significant upregulation of *CHOP* and *BAK* at mRNA levels by HAND1 (Figure 5C). These results indicated that HAND1 positively regulates the transcription of *CHOP* and *BAK*, to further regulate cell apoptosis.

## Discussion

The high morbidity and mortality of GC is generally caused by poor prognosis and limited treatment strategies 31. Epigenetic inactivation of TSGs promotes GC tumorigenesis and progression, with a series of TSGs already identified in GC 32, 33. In this study, HAND1, a transcription factor and cell differentiation regulator, is found silenced or downregulated frequently in GC due to its promoter CpG methylation. Consistent with our results, HAND1 has been reported to be silenced by methylation in other cancers including colorectal cancer 12-15, 17-19. *HAND1* methylation is closely correlated with poor prognosis in GC, especially for late-stage patients 14. In thyroid cancer, HAND1 has been confirmed to be downregulated in differentiated and undifferentiated carcinomas, but expressed normally in benign neoplastic lesions and normal thyroid, and HAND1 restoration inhibits tumor cell growth 18. HAND1 expression was also significantly associated with other clinicopathological features of GC patients, including depth of invasion, lymph nodal status, and TNM stage. In addition, we found that HAND1 restoration inhibited GC cell growth, proliferation and migration, suggesting that HAND1 does functions as a *bona fide* tumor suppressor in GC. This is consistent with previous reports that HAND1 expression suppresses uPAR-induced tumor growth and angiogenesis and inhibits tumor cell invasion and metastasis in medulloblastoma; and HAND1 acts as a tumor suppressor inhibiting colorectal cancer cell growth and xenograft tumor formation 20, 21, 23.

**Scheme 1:**
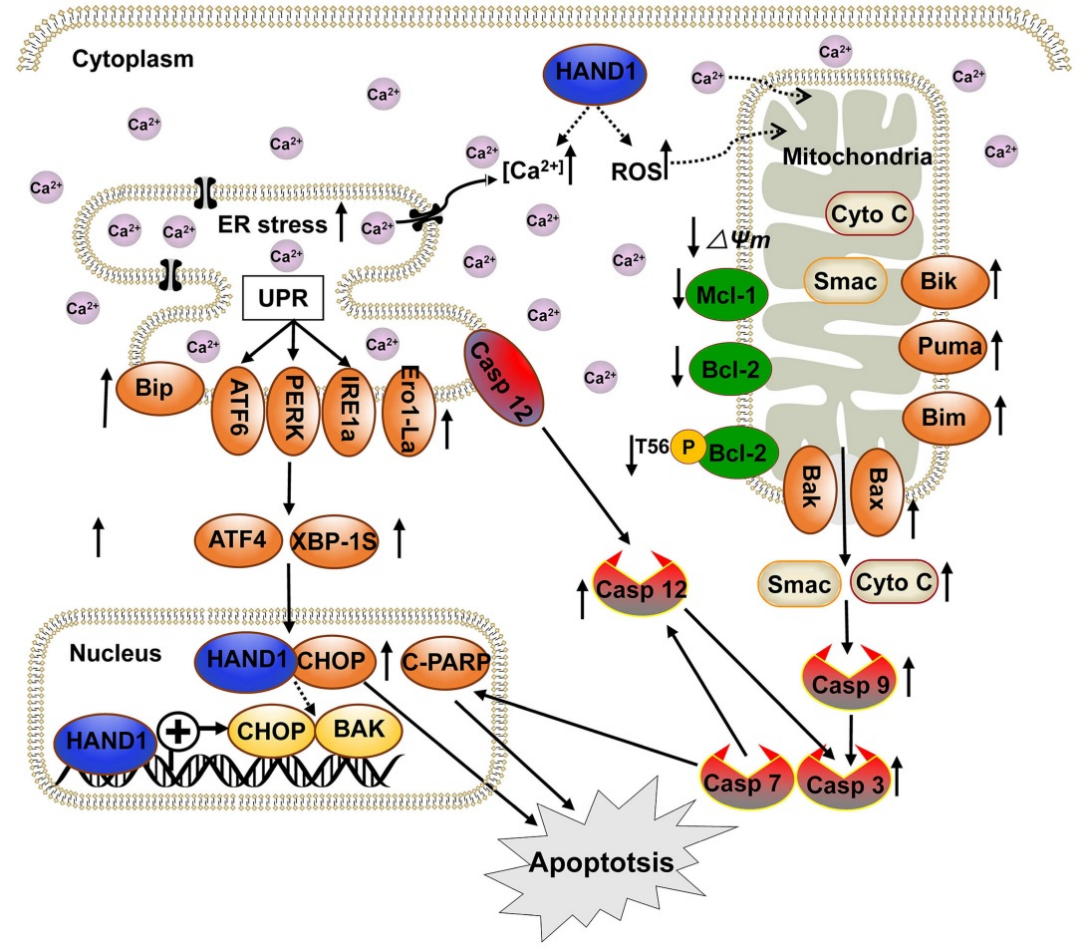
** Diagram showing the mechanism of HAND1 tumor suppression in gastric cancer.** HAND1 binds to CHOP and BAK promotors, upregulates their transcription, interacts with CHOP, and induces ER-stress mediated apoptosis including UPR and mitochondrial apoptosis via caspase-dependent pathway in gastric cancer cells. Dotted line indicates that mechanisms remain unclear. △Ψm, mitochondrial membrane potential.

Our present study found that HAND1 induced GC cell apoptosis and ER stress. Cisplatin, one of the most commonly used chemotherapeutic agents to treat solid tumors including GC, increases ROS generation and Ca^2+^ release and induces cell apoptosis through ER stress and mitochondrial pathways 34-36. Our findings showed that HAND1 enhanced cisplatin-induced apoptosis, intracellular ROS levels, and cytosolic Ca^2+^ concentrations. Various exogenous or endogenous factors, including ROS generation and disruption of Ca^2+^ homeostasis contributed to ER stress and further triggered UPR. UPR maintains ER function and reestablishes homeostasis, but if the stress occurs in excess or is sustained, then the apoptosis pathway is initiated 37, 38. This is consistent with our finding that HAND1 increased the expression of three major ER sensors, ATF6, PERK and IRE1a, as well as other UPR-related proteins such as Bip, ATF4 and XBP-1s. In addition, HAND1 expression upregulated CHOP, Ero1, and cleaved caspase-7, caspase-12 that are involved in ER stress-indued apoptosis (Scheme 1). CHOP is activated by three UPR-related pathways and upregulates Ero1, which induces cellular ROS generation and oxidative stress in ER, thereby contributing to apoptosis 39, 40. Our results confirmed that HAND1 elevates intracellular ROS levels in the absence of cisplatin. Caspase-12, which specifically resides on ER membrane, is activated by caspase-7 and released to cytosol in response to ER stress 41.

Dysregulated ER stress results in the transmission of ROS and Ca^2+^ signals from ER to mitochondria and triggers the response of BCL-2 family proteins, which then activates the mitochondrial apoptotic pathway. In this process, BH3-only proteins, which are activated by ER stress, inhibit anti-apoptotic BCL-2 family proteins and activate pro-apoptotic proteins Bax and Bak, leading to mitochondria outer membrane permeabilization (MOMP) and the release of cytochrome c and Smac 42. Our results suggested that HAND1 upregulates BH3-only proteins Bik, Bim and PUMA and pro-apoptotic protein Bax and Bak, whereas downregulates anti-apoptotic protein Bcl-2, p-Bcl2 (Thr56) and Mcl-1. Meanwhile, HAND1 expression led to the loss of MMP and release of Cyto C and Smac from the mitochondria to cytosol that further activated caspase-9 and caspase-3. Our findings showed that HAND1 induces ER-stress-mediated apoptosis, including UPR and mitochondrial apoptosis *via* the caspase-dependent pathway (Scheme 1). However, the mechanisms underlying the disruption of Ca^2+^ homeostasis and transmission of ROS and Ca^2+^ signals between ER and mitochondria mediated by HAND1 in GC still remain unclear and require further investigations.

CHOP, a marker for ER stress-induced apoptosis, is well known to upregulate pro-apoptotic protein Bax/Bak and increase translocation and oligomerization of Bax/Bak in ER 43. Bak, a key regulator of the intrinsic apoptosis pathway, is activated by cell stress and oligomerized on mitochondrial outer membrane leading to MOMP and cytochrome C release 44. CHOP and BAK are essential for the progression of cell apoptosis. HAND1 is a transcription factor that negatively or positively regulates the transcription of its target genes by binding to their promoter region 45-47. HAND1 targets *CHOP* and *BAK* promoters and positively regulates their transcription. Additionally, HAND1 interacts with CHOP, overexpression of CHOP increased BAK expression, and knockdown of CHOP or BAK attenuated HAND1-induced GC cell apoptosis, indicating that HAND1 induces GC cell apoptosis through CHOP and BAK. HAND1 regulates *CHOP* and *BAK* expression at the transcriptional level through interacting with CHOP, and further promotes GC tumor cells apoptosis and leads to tumor suppression.

## Conclusions

We have demonstrated that *HAND1* is frequently downregulated and methylated in GC. HAND1 functions as a tumor suppressor that induces ER-stress-mediated apoptosis including UPR and mitochondrial apoptosis by targeting CHOP and BAK in GC cells. Furthermore, we confirmed that HAND1 interacts with CHOP, and positively regulates *CHOP* and *BAK* transcription in GC (Scheme 1). We also showed that HAND1 promoter methylation is a potential prognostic epigenetic biomarker for GC patients.

## Supplementary Material

Supplementary tables.

## Abbreviations

GC: gastric cancer
HAND1: Hand-And-Neural-crest-Derivative-expressed 1
HAND: Hand-And-Neural-crest-Derivative-expressed
ROS: reactive oxygen species
UPR: unfolded protein response
TSG: tumor suppressor gene
bHLH: basic helix-loop-helix
CGI: CpG island
MSP: methylation-specific PCR
BGS: bisulfite genomic sequencing
5-Aza: 5-Aza-2'-deoxycytidine
TSA: trichostatin A
IHC: immunohistochemistry
OS: overall survival
MMP: mitochondrial membrane potential
RNA-Seq: RNA sequencing
Co-IP: Coimmunoprecipitation
MOMP: mitochondria outer membrane permeabilization
ER: endoplasmic reticulum
C-PARP: cleaved PARP
Cyto C: cytochrome c
siRNA: small interfering RNA
OE: overexpression
CHIP: chromatin immunoprecipitation
TSS: transcription start site
