# Supplementary material for: The developmental regulator HAND1 inhibits gastric carcinogenesis through enhancing ER stress apoptosis via targeting CHOP and BAK which is augmented by cisplatin
Source: Int J Biol Sci. 2023 Jan 1;19(1):120–36. doi: 10.7150/ijbs.76345 (PMC9760445; doi:10.7150/ijbs.76345)
Supplement: Supplementary file 1 — Supplementary tables. [file ijbsv19p0120s1.pdf]

Table S1. Correlation between HAND1 expression and clinicopathological parameters of GC patients

Clinicopathological parameters	N (%)	HAND1 expression		X^2	*p*-value
		Low (%)	High (%)		
**Total**	165	111 (67.3)	54 (32.7)		
**Gender**					
Male	120(72.7)	87 (72.5)	33 (27.5)	5.461	**0.019***
Female	45(27.3)	24 (53.3)	21(46.7)		
**Age**					
≥62	89(53.9)	65 (73)	24 (27)	2.913	0.088
＜62	76(46.1)	46(60.5)	30 (39.5)		
**Histopathological grading**					
Well / moderately	103(62.4)	75(72.8)	28 (27.2)	3.825	**0.050***
Poorly	62(37.6)	36 (58.1)	26(41.9)		
**Depth of invasion**					
pT1/T2	53(32.1)	29(54.7)	24(45.3)	5.591	**0.018***
pT3/T4	112(67.9)	82 (73.2)	30(26.8)		
**Lymph nodal status**					
N0	50(30.3)	27(54)	23 (46)	5.740	**0.017***
N1/2/3	115(69.7)	84(73)	31(27)		
**Distant metastasis**					
M0	135(81.8)	87 (64.4)	48(35.6)	2.698	0.100
M1	30(18.2)	24(80)	6 (20)		
**TNM stage**					
I	35(21.2)	15 (42.9)	20(57.1)	13.033	**0.005***
II	34(20.6)	23(67.6)	11(32.4)		
III	67(40.6)	50(74.6)	17(25.4)		
IV	29(17.6)	23(79.3)	6 (20.7)		

$^*p < 0.05$.

Table S2. Univariate survival analyses (Cox regression model) of various factors in GC patients

**Characteristics**	**Categories**	**HR**	**95% CI**	***p*-value**
Gender	Male/Female	0.945	0.588-1.517	0.813
Age	≥62/<62	0.571	0.368-0.885	**0.012***
Histopathological grading	Well/moderately/poorly	0.63	0.399-0.993	**0.047***
Depth of invasion	T1/T2/T3/T4	5.848	3.01-11.361	**< 0.001***
Lymph node metastasis	N0/N1/N2/N3	3.761	2.039-6.939	**< 0.001***
Distant metastasis	M0/M1	4.734	2.921-7.673	**< 0.001***
TNM stages	I/II/III/IV	2.613	1.996-3.422	**< 0.001***
HAND1 expression	Low/High	2.632	2.01-3.448	**< 0.001***

HR, hazard ratio; CI, confidence interval. $^{*}p < 0.05$.

Table S3. Multivariate survival analyses (Cox regression model) of prognostic factors in GC patients

**Characteristics**	**Categories**	**HR**	**95% CI**	***p*-value**
Age	≥62/＜62	0.744	0.47-1.178	0.207
Histopathological grading	Well/moderately/poorly	0.818	0.505-1.324	0.413
Depth of invasion	T1/T2/T3/T4	3.859	1.833-8.122	**< 0.001***
Lymph node metastasis	N0/N1/N2/N3	1.998	0.942-4.241	0.071
Distant metastasis	M0/M1	2.465	1.018-5.97	**0.046***
TNM stages	I/II/III/IV	1.139	0.643-2.019	0.656
HAND1 expression	Low/High	0.216	0.105-0.445	**< 0.001***

HR, hazard ratio; CI, confidence interval. *$p < 0.05$.

Table S4. PCR primers used in the study

Primers	Sequences (5'-3')
**For HAND1 RT-PCR**	
HAND1-F	CCAAGCTCTCCAAGATCAAG
HAND1-R	GTCCTTTAATCCTCTTCTCG
**Control for RT-PCR**	
GAPDH-F	GGCTCTCCAGAACATCATCCCTGC
GAPDH-R	GGGTGTCGCTGTTGAAGTCAGAGG
**For detecting methylation of HAND1 promoter by MSP**	
HAND1-M-F	TTTTTTTATCGGTTTTTGTCGC
HAND1-M-R	CTAACGAACTAAAATAACGCG
**For detecting unmethylation of HAND1 promoter by MSP**	
HAND1-U-F	GTTTTTTTATTGGTTTTTGTTGT
HAND1-U-R	TACTAACAAACTAAAATAACACA
**For BGS analysis of HAND1 methylation**	
HAND1-BGS-F	TATTTTTAGAGTTGGTTGTTGAGTT
HAND1-BGS-R	CTCCTTCTTAAATCCTAAACCTTTC
**For CHIP-PCR analysis of CHOP promotor region that HAND1 bound to**	
CHOP-CHIP-F	CTCACACAGGGAGTCACAGG
CHOP-CHIP-R	GTGGACCCGATTCTGTTTGG
**For CHIP-PCR analysis of BAK promotor region that HAND1 bound to**	
BAK-CHIP-F	CCACCCAGATCACCCTACAG
BAK-CHIP-R	GTTTCCAGGAATGGGCGTC
**For quantitative real time RT-PCR**	
GAPDH-F	TGCACCACCAACTGCTTAGC
GAPDH-R	GGCATGGACTGTGGTCATGAG
HSPA5-F	CGTCCTATGTCGCCTTCACT
HSPA5-R	TGTCTTTGTTTGCCCACCTC
ATF6-F	GCTCTCTTTGCTGAACTCGG
ATF6-R	TGAGGAGGCTGGAGAAAGTG
PERK-F	CGGAACCAGACGATGAGACA
PERK-R	TCCCACATCCAAATCCCACT
ATF4-F	GCCAAGCACTTCAAACCTCA
ATF4-R	GGTCATCTGGCATGGTTTCC
IRE1a-F	GTGTCAACGCTGGATGGAAG
IRE1a-R	ACAGGCTGCCATCATTAGGA
XBP-1-F	TGGTGCCAGCCCAGAGAG
XBP-1-R	CTGTTCCAGCTCACTCATTCG
Ero1-La-F	GAGACAGCGGCACAGAGGT
Ero1-La-R	CAGCACAGTCCCTTCTTCCA
CHOP-F	CATTGCCTTTCTCCTTCGGG

CHOP-R	CCAGAGAAGCAGGGTCAAGA
BAK-F	TAGCCCAGGACACAGAGGAG
BAK-R	ATAGCGTCGGTTGATGTCGT
